# Health Management Decision of Sensor System Based on Health Reliability Degree and Grey Group Decision-Making

**DOI:** 10.3390/s18072316

**Published:** 2018-07-17

**Authors:** Kai Song, Peng Xu, Guo Wei, Yinsheng Chen, Qi Wang

**Affiliations:** 1 School of Electrical Engineering and Automation, Harbin Institute of Technology, Harbin 150001, China; kaisong@hit.edu.cn (K.S.); weiguo@sina.com (G.W.); wangqi@hit.edu.cn (Q.W.); 2 School of Measurement and Communication Engineering, Harbin University of Science and Technology, Harbin 150001, China; chen_yinsheng@126.com

**Keywords:** health management decision, grey group decision-making, health reliability degree, maintenance decision, sensor system

## Abstract

Metal Oxide Semiconductor (MOS) gas sensor has been widely used in sensor systems for the advantages of fast response, high sensitivity, low cost, and so on. But, limited to the properties of materials, the phenomenon, such as aging, poisoning, and damage of the gas sensitive material will affect the measurement quality of MOS gas sensor array. To ensure the stability of the system, a health management decision strategy for the prognostics and health management (PHM) of a sensor system that is based on health reliability degree (*HRD*) and grey group decision-making (*GGD*) is proposed in this paper. The health management decision-making model is presented to choose the best health management strategy. Specially, *GGD* is utilized to provide health management suggestions for the sensor system. To evaluate the status of the sensor system, a joint *HRD*-*GGD* framework is declared as the health management decision-making. In this method, *HRD* of sensor system is obtained by fusing the output data of each sensor. The optimal decision-making recommendations for health management of the system is proposed by combining historical health reliability degree, maintenance probability, and overhaul rate. Experimental results on four different kinds of health levels demonstrate that the *HRD*-*GGD* method outperforms other methods in decision-making accuracy of sensor system. Particularly, the proposed *HRD*-*GGD* decision-making method achieves the best decision accuracy of 98.25%.

## 1. Introduction

Sensor systems are extensively used in many fields, such as industry, manufacturing, aviation, and aerospace. Metal Oxide Semiconductor (MOS) gas sensor has become the most common gas sensor in sensor system at present because it has the advantages of fast response to target gas, high sensitivity, simple structure, easy to operation, low cost, and so on. Limited to the properties of metal oxide gas sensitive materials, the phenomenon such as aging, poisoning and damage of the gas sensitive material will affect the measurement quality of MOS gas sensor array. As a result, the trained pattern recognition method greatly degrades the performance of odor detection and analysis to target gases [1]. The influence of the work state and measurement quality of MOS gas sensor array to the performance of sensor system cannot be ignored.

At present, the following three ways are used to improve the fallen performance of the odor detection and analysis for sensor system that is caused by the decrease of reliability of the measurement value of the MOS gas sensor array.

(1) Improve the material, structure and technology of gas sensor to optimize the stability of gas sensor [2,3].
(2) Take high redundancy gas sensor array for data acquisition to minimize the impact of fault sensor on the detection and analysis effect of pattern recognition methods subsequently [4].
(3) Adopt periodic calibration and maintenance for sensor to replace the gas sensor whose performance decrease obviously [5,6].

Although the above methods can improve the reliability of sensor system to some extent, there are their own application limitations still existing. Due to the inherent characteristics of MOS gas-sensing materials, the current technology cannot completely solve the problem of stability of gas-sensing materials. The high redundancy gas sensor array can only reduce the influence of the fault sensor, but it cannot completely eliminate the effect of fault gas sensor on the detection and analysis results of the sensor system. Regular calibration and maintenance not only consume a lot of manpower and material resources, but they also cannot determine the working state and measurement quality of the sensor system during the period between twice calibration and maintenance consequently [7,8,9,10]. There are numerous sensitive elements and components in a sensor system and relationships among certain components that influence each other. Sensitive elements and components often exposed to harsh environments (high temperature, high pressure, and strong corrosion), which cause the system to fail. In the past, when one or several sensors faulted in sensor arrays, changing the failure sensors is often applied. However, it is difficult to guarantee that the consistency of sensors is exactly all the same in replacement. It is necessary to rectify the parameters of the concentration output model. Sometimes, there are no standby sensors when the sensor is failure. Therefore, it is necessary to apply health management decision-making to the sensor system. To ensure the stability of the system, a suitable solution must be determined to make the health status of all the sensors in the system more clear.

**Figure 1:**
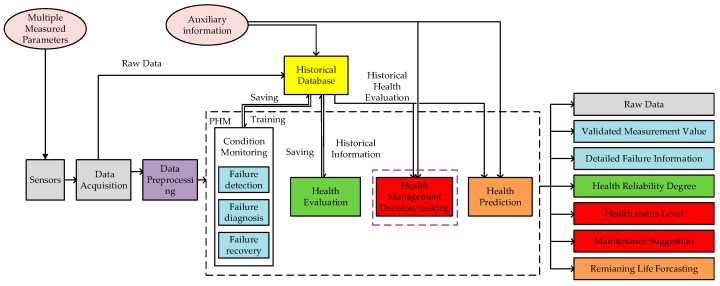
Prognostics and health management (PHM) structure of sensor system.

Prognostics and health management (PHM) is used widely in a great number of fields [11,12,13]. Prognostics and health management decision is a synthesis technique that includes data acquisition, failure detection, failure diagnosis, failure recovery, health evaluation, failure prediction, maintenance decision-making, and any other aspect [14,15,16]. The purpose of health management decision is to improve the safety and reliability of systems. Health management decision can achieve an evaluation and prediction of system health status according to the collected data [17]. According to the health management decision-making method, maintenance recommendations are provided. In other words, choosing the corresponding measures to reduce the failure level or to prevent the occurrence of a fault. In this way, system state is clearer and the maintenance times are reduced accordingly [18,19,20]. The PHM structure of sensor system is shown in Figure 1. Based on some previous work [21,22,23,24,25,26,27], the research of condition monitoring and health evaluation has been completed. This paper will focus on health management decision-making on the basis of condition monitoring and health evaluation methods. The purpose of this paper is not to separate the other parts of PHM from health management decision-making, but to increase the reliability of the system combined with the other parts.

The current health management decision method, according to the theory and technical application in research, can be divided into three categories: model-based maintenance decision-making [28], data-based maintenance decision-making [29,30] and reliability-based decision-making [31,32].

The sensor system has a complex structure and changeable working condition and is easily affected by the environment. The outputs of sensors are greatly influenced by the environment. The baselines of the same concentrations are different at different times. It is difficult to define the failure range. As a result, it is hard to build an appropriate model for maintenance decision-making of sensor system. Data-based maintenance decision-making method is difficult to build for the same reason. The traditional reliability-based method, such as D-S evidence theory [33,34,35,36], Bayes theory [37,38], and fuzzy set theory [39,40,41,42,43], will face severe challenges with the uncertainty of information and variety of data types. When provided with conflicting evidence, the D-S evidence theory results tend to deviate from the understanding of the user. Under system failure, using D-S evidence theory to meet the conflict changing from health status to failure status is difficult. Priori probability is essential for the Bayes decision method. Accuracy results are easily obtained when priori probability is known. However, obtaining priori probability is difficult. The application will be limited to some extent. Fuzzy set theory is a great data fusion method, but when handling maintenance decision-making, there are many subjective factors in the description of information because of its logical reasoning. Therefore, the representation and processing of information lacks objectivity. The three types of maintenance decision-making methods are difficult to apply for such systems. In order to evaluate the work status of the sensor, Shen et al. proposed the concept of health reliability degree of multi-functional sensors [25]. Health Reliability Degree (*HRD*) is a quantitative description of heath information. However, when there are too many sensors, a single sensor failure cannot be effectively reflected [26].

In recent years, group decision-making technology has rapidly developed [44,45,46,47]. The main research content of group decision-making is making effective decisions when multiple decision makers make decisions simultaneously. The main problem that must be solved is how to aggregate the decision information of different experts with different preferences to obtain consistent decision results. By fusing the decision objective of each expert, the accuracy of the system can be improved. However, group decision-making technology research is undeveloped. The methods of correctly obtaining decision information, including property value, property weight, and decision maker weight information have not been established.

In this paper, a method for health management and maintenance decision based on health reliability degree and grey group decision-making (*HRD*-*GGD*) is proposed. In this method, *HRD* of sensor system is obtained by fusing the output data of each sensor. The optimal decision-making recommendations for health management of the system is proposed by combining historical health reliability degree, maintenance probability, and overhaul rate. The *HRD*-*GGD* is proposed to realize the maintenance of the sensor system by comprehensively considering the decision results of multiple expert sets. Not only can the system give out the system state, but also provide the maintenance suggestion for each failure mode after the system working and give the confidence degree of each maintenance proposal.

The rest of this paper is organized as follows. In Section 2, the framework of health management and maintenance decision and the corresponding methods are presented. In Section 3, the experimental setup and analytical discussion are introduced. In Section 4, two situations are presented and 400 different health status level samples are analyzed to give the results of health management decision. Finally, the conclusion is accounted in Section 5.

## 2. Health Management Decision

### 2.1. Implementation Framework of Health Management and Maintenance Decision

The main purpose of the health management decision is to obtain the working state of sensor system quantitatively and to provide the maintenance decision for the system at this status. In order to evaluate the state of the sensor system, the historical failure information, historical maintenance records, and trends of historical health status for the system is used in order to model the health management mode. As the state of the system is clearer, it is easy to reduce the proportion of unscheduled maintenance in the maintenance plan and change the unscheduled maintenance to predictive maintenance (scheduled maintenance).

**Figure 2:**
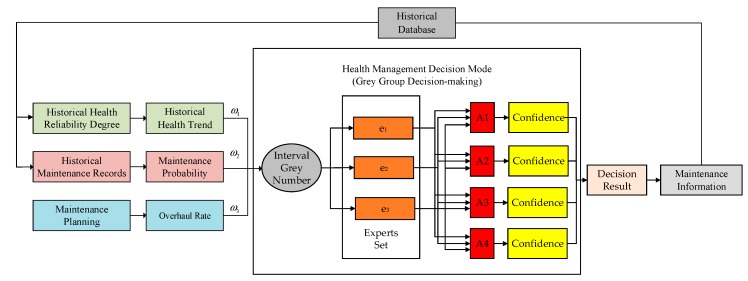
Framework of the health management decision.

The establishment of framework is the core of health management decision theory. The health management suggestion is dynamically obtained by collecting fault information, health status, failure prediction conclusion, and historical maintenance situation. The framework is shown in Figure 2. The system input vector is composed of three parameters: historical health trend, maintenance probability, and overhaul rate. The historical health trend indicates the working state of the system during the last period of time. The parameter is acquired by fusing the historical *HRD* during this period. Maintenance probability is obtained from historical maintenance records. The value is equal to history maintenance times/total test times. The more frequent maintenance of the system, the greater the probability of failure. The value of maintenance probability is larger at this condition. Overhaul rate is the parameter of unpredictable maintenance task. The value is equal to the next inspection time/overhaul cycle. The longer the overhaul time, the greater the uncertainty of the system. The system is inclined to failure in this way.

There are three experts in the expert set. The experts can be changed when facing different problems. In the system, three algorithms are used as the experts, namely D-S evidence theory, Bayes theory, and fuzzy set theory. Three experts give their suggestion, respectively, according to the above three parameters. It can be found from the experiment that these three methods have their limitations, respectively, which will be discussed in Section 4.

The second part is the group decision-making. This part is responsible for data fusion of the decisions of the expert set to obtain the final decision result. The decision information is recorded as part of the next decision. The decision information is recorded and used as the basis for the next decision. The solution set of grey group decision-making is $\left\{A_{1},A_{2},A_{3},A_{4}\right\}$, the corresponding decision frameworks are *A*_1_ {no maintenance}, *A*_2_ {preventive maintenance}, *A*_3_ {corrective maintenance}, and *A*_4_ {immediate maintenance}. The decision expert set is $\left\{e_{1},e_{2},e_{3}\right\}$, which represent three experts, respectively. The decision index set is $\left\{u_{1},u_{2},u_{3}\right\}$. The corresponding evidences are *u*_1_ {historical health reliability degree}, *u*_2_ {unpredictable maintenance task}, and *u*_3_ {historical maintenance record}. The evidence property weight vector for three evidences is $\omega =\{\omega_{1},\omega_{2},\omega_{3}\}$. The size of decision framework is four. The decision framework and its corresponding health status levels and maintenance levels are shown in Table 1.

The state description and corresponding maintenance suggestion are shown, as follows:

1. Health: The whole system is very healthy. All of the sensors are also healthy. Their measurements are close to the expected value. There is no need to repair the system.
2. Subhealth: The system is working at subhealthy status. The output of the system is within a normal range. All of the parameters may fluctuate near their expected value. It is essential to execute preventive maintenance regularly. Failure detection and failure isolation methods should be used in this situation.
3. Failure Edge: The system is nearly failure. Their actual measurements have deviated from the expected value, but they have not deviated completely. In this status some sensors may be faulty, but the system can work effectively when fault recovery is performed. Corrective maintenance is needed after experiment [25]. Failure recovery method will be applied in this status to improve the work status sometimes.
4. Failure: The system is failure. Most of sensors are failure. The actual output has completely deviated from its expected results. Immediate repaired the failure components or replacement failure components immediately may be the best choice.

Maintenance decision method can provide the maintenance suggestion for each failure mode and give the confidence degree of each maintenance proposal.

### 2.2. Health Reliability Degree (HRD)

*HRD* is a novel conception to define a quantitative health level. *HRD* represents the health level of whole system. The *HRD* of the system is fused by the health level of all the sensitive elements in the system. The value ranges from 0 to 1. When the value is 0, the system works at a severe failure state. When the value is 1, the system works at 100% healthy state. The larger the *HRD*, the higher the health level. The relationship between *HRD* and health level is defined as Table 2. When to evaluate the health status of the system, the four health status levels, healthy status (*HS*), subhealthy status (*SHS*), failure edge status (*FES*), and failure status (*FS*). The specific values vary according to different application objects [26].

**Figure 3:**
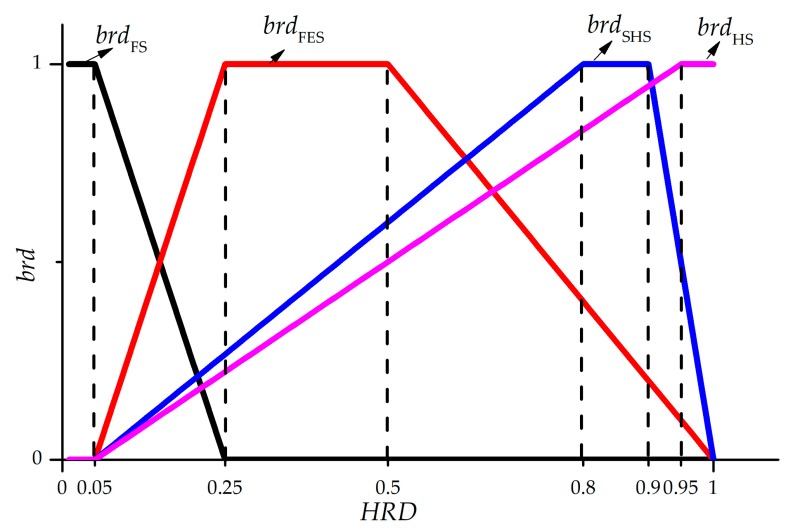
The relationship between Health Reliability Degree (*HRD)* and Belonging Relationship Degree (*brd*).

*HRD* is fused of four belonging relationship degree (*brd*) of sensor system by applying grey theory. The four parameters *brd* are the keys to computation *HRD*. The values can be expressed in a simplified way, as shown in Figure 3. If the *brd* is equal to 1, then the current working status is completely belonged to its corresponding status completely. If the *brd* is equal to 0, then the current status is completely not belonged to its corresponding status. The *brd* is changed with the fluctuation of *HRD*. In order to map the relationship between *HRD* and *brd*, Relevance Vector Machine (RVM) is used to fuse four *brd* to *HRD*.

**Figure 4:**
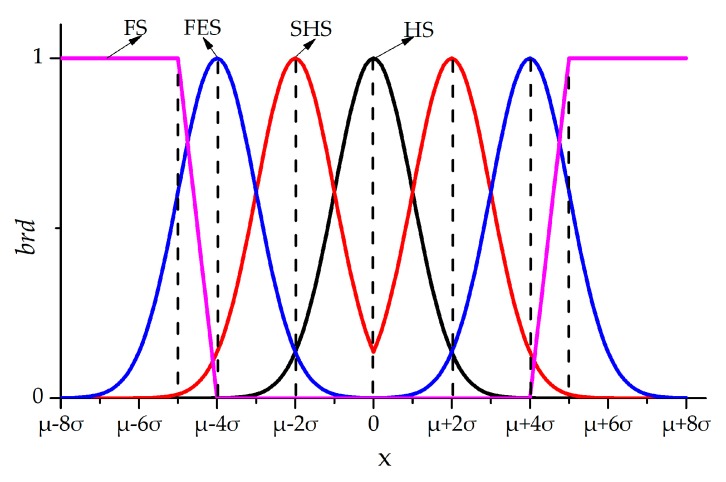
The relationship between the output and *brd*.

The relationship of *brd*s and output parameters are shown in Figure 4. In summary, the whitening function of four grey sets are obtained by Equations (1)–(4).

(1) $$f_{HS}\left(x\right)=\exp\left[-{\left\|x-\mu \right\|}^{2}/2\delta^{2}\right]$$

(2) $$f_{SHS}\left(x\right)=\left\{ \begin{array}{l} \begin{array}{cc} \exp\left[-{\left\|x-\mu +\delta \right\|}^{2}/2\delta^{2}\right] & x<\mu \end{array}\\ \begin{array}{cc} \exp\left[-{\left\|x-\mu -\delta \right\|}^{2}/2\delta^{2}\right] & x>\mu \end{array} \end{array}\right.$$

(3) $$f_{FES}\left(x\right)=\left\{ \begin{array}{l} \begin{array}{cc} \exp\left[-{\left\|x-\mu +3\delta \right\|}^{2}/2\delta^{2}\right] & x<\mu \end{array}\\ \begin{array}{cc} \exp\left[-{\left\|x-\mu -3\delta \right\|}^{2}/2\delta^{2}\right] & x>\mu \end{array} \end{array}\right.$$

(4) $$f_{FS}\left(x\right)=\{\begin{array}{ll} 1 & \begin{array}{ccc} x<\mu -5\delta & or & x> \end{array}\mu -5\delta \\ -1/\delta \cdot \left[x-\mu +4\delta \right] & \mu -5\delta <x<\mu -4\delta \\ 1/\delta \cdot \left[x-\mu -4\delta \right] & \mu +4\delta <x<\mu +5\delta \\ 0 & \mu -4\delta <x<\mu +4\delta \end{array}$$

For the all of the components in sensor system, the grey sample evaluation (*GSE*) matrix at the single time point can be expressed as $GSE_{j}={\left(gse_{ijk}\right)}_{m\times n}\left(i=1,2,\cdots ,m;k=1,2,\cdots ,n\right)$, which is shown in (5).

(5) $$GSE_{j}=\begin{array}{cc} & \begin{array}{cccc} I_{1}\; & \;I_{2} & \;I_{3} & \;I_{4} \end{array}\\ \begin{array}{c} S_{1}\\ S_{2}\\ \vdots \\ S_{m} \end{array} & \left[\begin{array}{cccc} a_{1j1} & a_{1j2} & a_{1j3} & a_{1j4}\\ a_{2j1} & a_{2j2} & a_{2j3} & a_{2j4}\\ \vdots & \vdots & \vdots & \vdots \\ a_{mj1} & a_{mj2} & a_{mj3} & a_{mj4} \end{array}\right] \end{array}$$

where *j* represents the time point, *S*_1_ indicates all the elements in sensor system. *I_k_* is the evaluating criterion.

The decision weight vector of different elements in sensor system is obtained by using information entropy method. The probabilistic proportion of *k*th assessment criterion of *i*th elements is shown in (6).

(6) $$\begin{array}{cc} P_{ik}=\frac{gse_{ik}}{\sum_{k=1}^{n}gse_{ik}} & \left(i=1,2,\cdots ,m\right) \end{array}$$

Then compute the information the information entropy of the *i*th elements by (7).

(7) $$\begin{array}{cc} E_{i}=-\frac{1}{\ln n}\sum_{k=1}^{n}P_{ik}\ln P_{ik} & \left(i=1,2,\cdots ,m\right) \end{array}$$

The weight vector $W_{j}=\{w_{1j},w_{2j},\cdots ,u_{mj}\}$ is determined by

(8) $$\begin{array}{cc} w_{ij}=\frac{1-E_{i}}{\sum_{i=1}^{m}\left(1-E_{i}\right)} & \left(i=1,2,\cdots ,m\right) \end{array}$$

After obtaining the decision weight, the comprehensive grey assessment values (*CGAV*) are calculated by (9).

(9) CGAV=W×GSE

where $CGAV=[\begin{array}{cccc} brd_{SH} & brd_{SHS} & brd_{FES} & brd_{FS} \end{array}]$.

**Figure 5:**
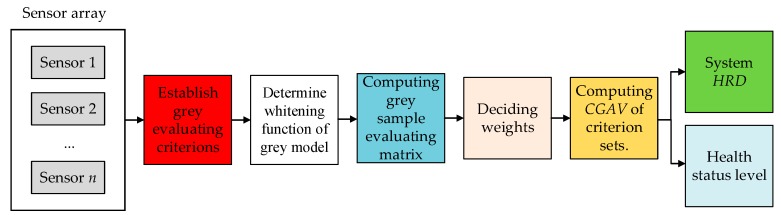
The flowchart of *HRD* methodology.

The flowchart of the *HRD* methodology is shown in Figure 5 and the detail steps are shown in Table 3. The correlation among multiple parameters has been fully considered for the weight distribution of different sensors.

### 2.3. Grey Group Decision-Making

#### 2.3.1. Grey Risk Decision-Making

The scheme-set of grey risk group decision-making problem is $\left(A_{1},A_{2},\ldots ,A_{n}\right)$, the decision indicator set is $\left(u_{1},u_{2},\ldots ,u_{m}\right)$, and the decision group set is $\left(e_{1},e_{2},\ldots ,e_{q}\right)$ (q≥2), where $e_{s}$ represents the *s*-th decision maker. For each decision indicator $u_{j}$, there are *l* possible states $\theta =\{\theta_{1},\theta_{2},\ldots ,\theta_{l}\}$. The probability of the state $\theta_{t}$ occurring is $p_{tj}^{s}(1\leq t\leq l)$ for the decision maker $e_{s}$ under the decision indicator $u_{j}$, which fits $\left(0\leq p_{tj}^{s}\leq l\right)$, $\sum_{t=1}^{l}p_{tj}^{s}=l$. The attribute value of plan $A_{i}$ is $a_{ijt}^{s}(⊗)\in [{\underline{a}}_{ijt}^{s},{\bar{a}}_{ijt}^{s}]$ [48]. The expert decision-making is divided into *f*-levels. The grey expert attribute set is denoted as $M=\left\{A_{1},A_{2},\ldots ,A_{f}\right\}$.

For the decision expert attribute set *M* of level *f*, the decision expert fuzzy attribute value is $m_{s}=k(1\leq k\leq f)$. Define the deviation coefficient $a_{s}$, which indicates the difference between $m_{s}$ and the *s*-th expert actual importance of the expert and $a_{s}\in [-0.5,0.5]$.

The index $\omega =(\omega_{1},\omega_{2},\ldots ,\omega_{m})$ weight is obtained while using the reciprocal of entropy weight method to increase the experts weight with higher accuracy. If the information entropy index is smaller, the more information it provides. The greater the role that it plays in the comprehensive evaluation, the greater the weight of the index. In order to reduce the effluence, the reciprocal was used in this way. The weight of the decision-maker *s* is

(10) $$H_{s}=\sum_{j=1}^{m}\omega_{j}\sum_{t=1}^{l}{\left(-p_{tj}^{s}\ln p_{tj}^{s}\right)}^{-1}\;$$

where $p_{tj}^{s}$ is the input data. Deviation factor is

(11) $$a_{s}=\frac{H_{s}-0.5(\min H_{s}+\max H_{s})}{\min H_{s}-\max H_{s}}$$

where $a_{s}\in [-0.5,0.5]$. Let $H_{s}=\min H_{s}$, $a_{s}=0.5$. If $H_{s}=\max H_{s},\;a_{s}=-0.5$. Therefore, the decision-making expert attributes after recuperation are $m_{s}^{\prime }=m_{s}+a_{s}$.

The expert weight is obtained, as follows:

(12) $$\lambda^{s}=\frac{m_{s}^{\prime }}{\sum_{s=1}^{q}m_{s}^{\prime }}$$

As *H_s_* increases, more right information included in the decision maker *s* increases the effectiveness and increases the weight. Thus, it is more realistic to correct the importance of the decision maker using entropy to obtain the decision maker weights.

Let $z_{ij}(⊗)=\sum_{s=1}^{q}r_{ij}^{s}(⊗)\lambda^{s}$, the standardized synthesis decision matrix can be obtained [49].

(13) $$z(⊗)={\left(z_{ij}(⊗)\right)}_{n\times m}={\left(\left[{\underline{z}}_{ij},{\bar{z}}_{ij}\right]\right)}_{n\times m}$$

#### 2.3.2. Grey Group Decision Model for Decision-Making

**Figure 6:**
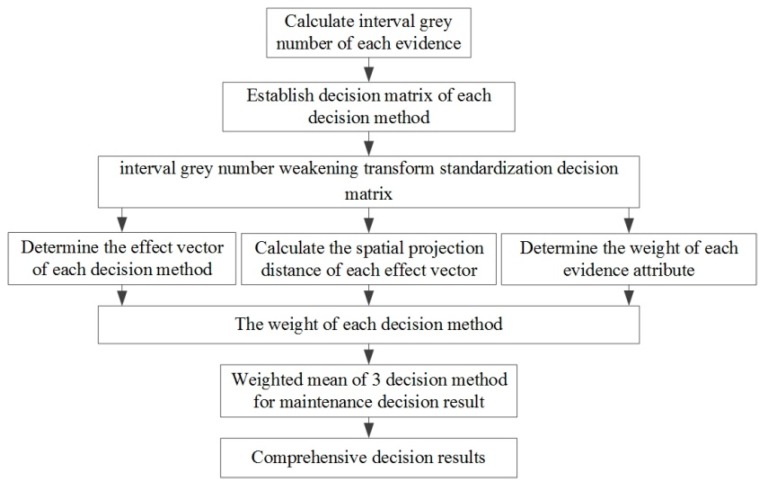
Decision diagram of grey group decision-making theory.

The grey group decision-making theory is used to make the effective choice of the three decision algorithms and the final comprehensive decision is made by fusing the three types of algorithms. The specific process is shown in Figure 6. The comprehensive step of the grey group decision-making theory is shown in Table 4. In summary, the maintenance decision results and the comprehensive confidence of the system failure modes can be obtained while using the grey group decision-making method, and the dynamic maintenance plan can be formulated on this basis.

### 2.4. The Process of Health Management Decision Method Based on Grey Group Decision

In order to improve the accuracy of the evaluation. The health management decision method combines grey group decision making and *HRD* theory to implement health management decision to sensor system. The detail steps of *HRD*-*GGD* are shown, as follows.

**Step 1:** To obtain the system measurement point parameters of the sensor or network interface, the data acquisition device is used to collect data.

**Step 2:** Pre-process the parameters collected by each measuring point in the system. Pre-processing includes abnormal value elimination, filtering noise reduction, calculating average value, and 3σ standard deviation.

**Step 3:** Failure detection, isolation, and diagnostic algorithms should be applied to determine the location of and the type of failure [21,22].

**Step 4:** If there is no failure in the array, give out the best estimation value of each sensor, according to the correlation of sensor array. If failure occurs in the sensor array, the best estimation value of failure sensor can be obtained, according to the normal data and the value of normal sensor can be got from the best estimation value of failure sensor and the other normal sensors.

**Step 5:** Establish the health reliability degree evaluation mode. The results of *HRD* are used as the inputs of the historical health status trend. The calculation steps of *HRD* are thoroughly introduced in Section 2.2.

**Step 6:** Establish a health management decision model based on grey group decision-making theory. Make maintenance decision on various failure modes and give out the corresponding maintenance suggestion.

Firstly, obtain the evidences for each expert.

The number of evidence is three, expressed as *u*_1_, *u*_2_, *u*_3_, known, as follows:

*u*_1_: Analysis of health reliability degree and historical health reliability degree;

*u*_2_: Historical failure information and corresponding maintenance records; and,

*u*_3_: Prepared system maintenance program.

**Figure 7:**
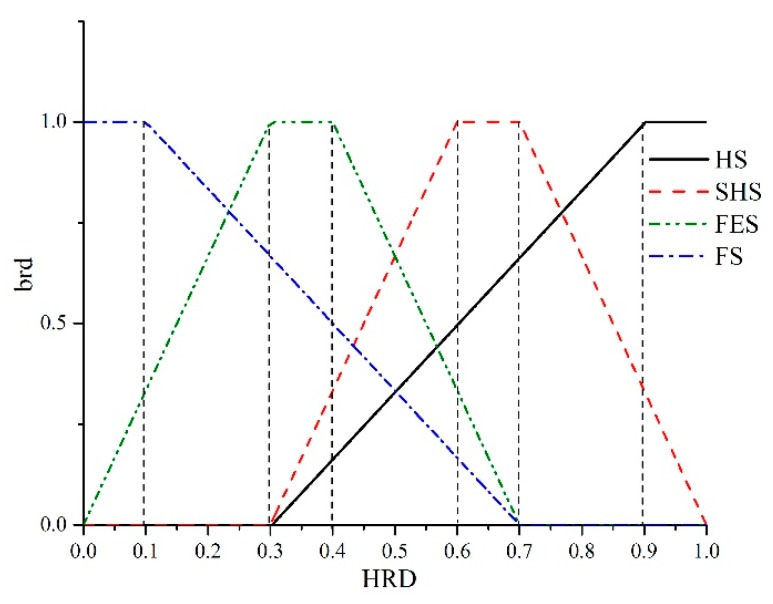
Whitening function of evidence *u*_2_ and *u*_3_.

Evidence *u*_1_ is fused according to three decision methods. Evidence u_2_ and u_3_ is obtained by using the whitening function as shown in Figure 7. *brd* is grey parameters. *HS* represents Health Status. *SHS* represents Subhealth Status. *FES* represents Failure Edge Status and *FS* represents Failure Status Level.

The size of the decision framework is 4. The framework is expressed with a 4-bit binary number:

*A*_1_: no maintenance, 0001,

*A*_2_: preventive maintenance, 0010,

*A*_3_: corrective maintenance, 0100, and

*A*_4_: immediate maintenance, 1000.

Utilizing the historical experiment data in Step 4. Analysis and prediction of the trend of the historical health parameters is based on the set of data. Determine the weight of each evidence attribute ω = (ω_1_, ω_2_, ω_3_) based on historical failure information and maintenance records combined with the prepared maintenance program. ω_2_ indicates that maintenance probability is equal to history maintenance times/total test times. ω_3_ is the overhaul rate, which is equal to the next inspection time/overhaul cycle. ω_1_ = 1 − ω_2_ − ω_3_.

Secondly, to process the input data and turn the data to grey numbers. Calculate the confidence intervals of each evidence with a significance level of 0.05.

Thirdly, establish the comprehensive decision matrix (CDM) of each decision method. The CDM of the three experts is calculated for each failure model. Then, the decision matrix is standardized to calculate the standardization comprehensive decision matrix. Finally, the final comprehensive decision results are obtained. The matrix includes upper bound evaluation matrices, lower bound evaluation matrices, and whitening evaluation matrices. The upper bound evaluation matrices are ${\mathrm{CDM}}^{\left(1+\right)}$, ${\mathrm{CDM}}^{\left(2+\right)}$, and ${\mathrm{CDM}}^{\left(3+\right)}$. The lower bound evaluation matrices are ${\mathrm{CDM}}^{\left(1-\right)}$, ${\mathrm{CDM}}^{\left(2-\right)}$, and ${\mathrm{CDM}}^{\left(3-\right)}$. The whitening evaluation matrices are ${\mathrm{CDM}}^{\left(1\right)}$, ${\mathrm{CDM}}^{\left(2\right)}$, and ${\mathrm{CDM}}^{\left(3\right)}$. The upper bound evaluation matrices represent the maximum confidence of three evidences in the system. The lower bound evaluation matrices represent the minimum confidence of three evidences in the system. Whitening evaluation matrixes represent the confidence of three evidences for whitening degree in the system. The matrices are shown as (14):

(14) $${\mathrm{CDM}}^{\left(k\right)}=\left[\begin{array}{cccc} \sigma_{11} & \sigma_{12} & \sigma_{13} & \sigma_{14}\\ \sigma_{21} & \sigma_{22} & \sigma_{23} & \sigma_{24}\\ \sigma_{31} & \sigma_{32} & \sigma_{33} & \sigma_{34} \end{array}\right]$$

where *k* represents the expert (*k* = 1, *e*_1_; *k* = 2, *e*_2_; *k* = 3, *e*_3_). $\sigma_{ij}$ represents the confidence of each evidence. *i* is the health level of the decision framework (*i* = 1, *A*_1_; *i* = 2, *A*_2_ ; *i* = 3, *A*_3_; *i* = 4, *A*_4_). *j* is the evidence (*j* = 1, historical health trends; *j* = 2, maintenance probability; *j* = 3, overhaul rate).

The steps to calculate the health status level using CDM are shown in Table 6.

Finally, ranking all of the alternative health status levels in accordance with the confidence and choosing the optimal health management suggestion with largest confidence.

## 3. Experimental Setup and Analytical Discussion

The detailed *HRD*-*GGD* process is given in Section 2.4. In this part, the problem of health management decision for an atmosphere pollution gas sensor system is taken as an example to verify the *HRD*-*GGD method*.

### 3.1. Sensor System Experimental System

**Figure 8:**
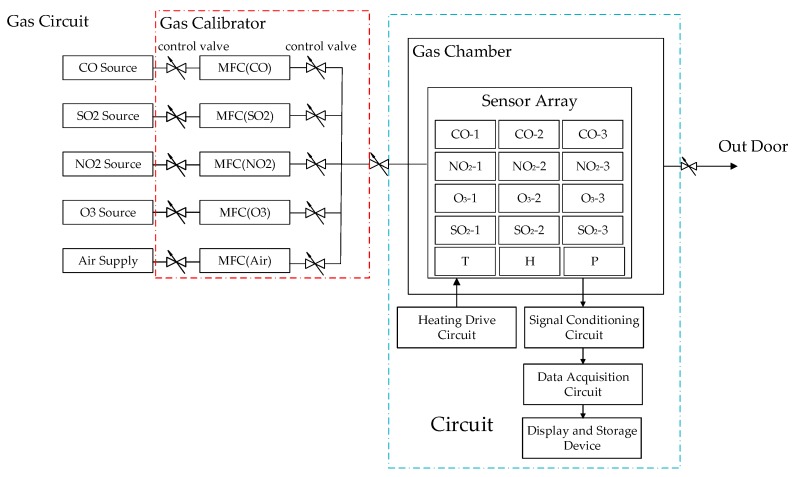
The model of the sensor system.

**Figure 9:**
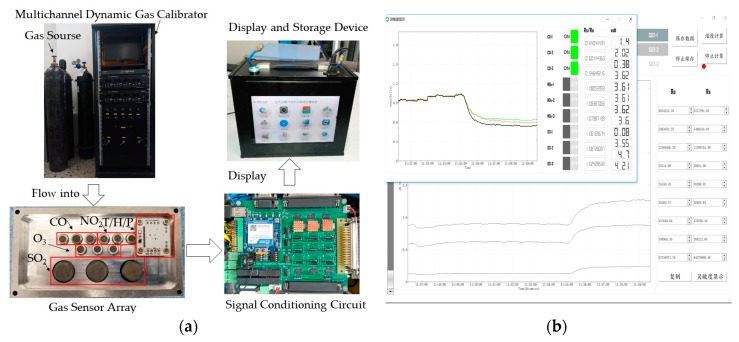
(**a**) Physical picture of sensor system; and, (**b**) display interface.

The sensor system used for testing atmosphere pollution gas was mainly composed of gas source, MFCs, gas chamber, sensor array, heater driver circuit, signal conditional circuit, data acquisition circuit, power supply, and laptop PC. The sensor array that consisted of four different types of gas sensors (CO, NO_2_, O_3_, SO_2_) and temperature, humidity, and pressure sensors, was fixed in the gas chamber. The number of each type gas sensor is three. The gas chamber temperature was maintained at 30 °C by constant temperature control. The structure of sensor system is shown in Figure 8 and the physical picture of sensor system is shown in Figure 9. The normal working ranges and units for the 15 sensors (three CO sensors, three NO_2_ sensors, three O_3_ sensors, three SO_2_ sensors, one temperature, one humidity, and one pressure sensor) are given in the Table 7. The health management decision platform is applied to the system. In the experiment, QT is used as the experimental software platform combined with SQL server software to realize database storage function. The *HRD*-*GGD* algorithm is implemented using Visual Studio.

The system is used in the laboratory. The acquisition device utilized USB-bus cards (USB-2089, Art Technology Development Co. Ltd., Beijing, China) with 16 analog inputs at up to 400 KHz and a 14-bit A/D conversion accuracy. The sampling period is once per second. The failure data is generated by the failure simulating software. By analysis of the feature of historical failure data, the failure forms of different failures are summarized in the software. The maintenance suggestions are given according to practical experience. The failure information and maintenance suggestions are given in Table 7.

### 3.2. Experiment Data

**Figure 10:**
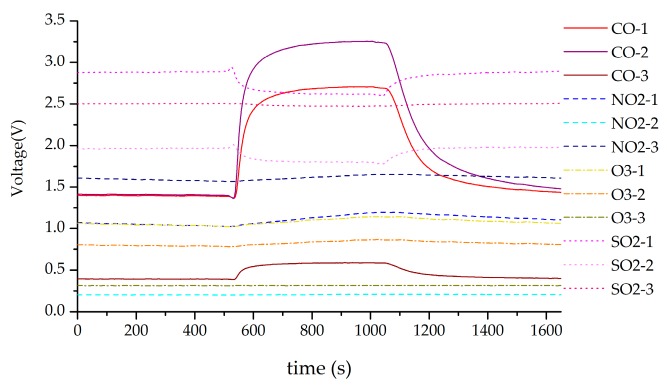
Response of the sensor system for 50 ppm CO.

**Figure 11:**
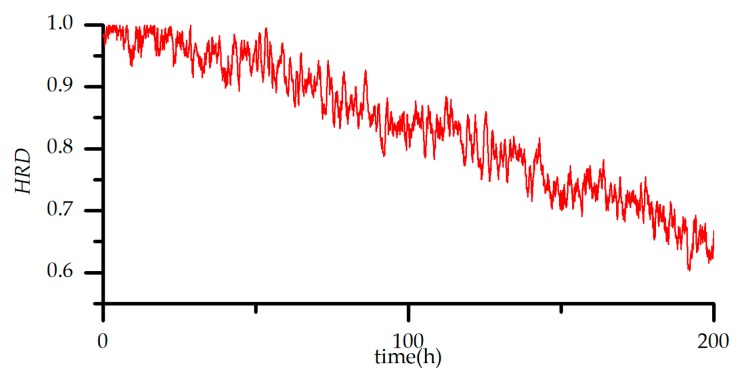
The historical *HRD* of the sensor system.

The data of each sensor are the voltage values collected by the 15 sensors in Table 8. Both the test and training samples include normal samples and fault samples. The normal samples are historical experimental data, i.e., system history experimental samples. The major frequent failure part in the sensor system is the sensor array. So, the failure type is mainly aimed at sensor array and heater drive circuit. The system *HRD* is the *HRD* of sensor array. The fault samples are analysed using fault simulation software, according to the fault modes. Figure 10 illustrates the sensor response process when exposed to 50 ppm CO in experiment. Each set of data includes 15 measurement points, with a sampling time of 1 s. The experiment involved 2 min for sensor to response completely and 2 min for the sensor to recover. When the system works for a long time, the performance of the sensor system will decrease with the increasing of running time. The historical *HRD* of the sensor system in 200 h is shown in Figure 11.

F1–F5 are the failure of sensors, F6 is the failure of heater circuit. Because of the correlation among components, the output tends to be abnormal when the system fails. F1 to F6 are both sensors or circuit faults for single sensor. Every sensor fault can be diagnosed as a kind of failure. Due to the same form of expression and different location, it can be classified as a kind of failure. When multiple failures occur, which is to say that different sensors have different failures at the same time. This situation can be understood as multiple fault superposition, not as new failure. 200 sets of historical data are used for obtaining the best work state. 400 groups data of different health status level (each type of health status level contains 100 groups data) are used for testing.

## 4. Results and Analysis

The health management decision of sensor system means that the maintenance suggestion is implemented in a global way and it refers to all of the sensors and components. In this section, two situations that represent different health levels are introduced to interpret the proposed strategy.

**Situation 1: all the sensor and components are fault free**

The collected data is processed using the grey data fusion method to obtain the historical health reliability degree, the details are shown in Section 2.2. The last seven health reliability degrees are used as the reference historical health parameter. The analysis and prediction of the historical health parameters trend are performed based on a data set. Historical failure information, maintenance records, and the established maintenance program are also considered.

Consider a random experiment as an example. The size of decision framework is 4. The framework is expressed as: {no maintenance}: *A*_1_, {preventive maintenance}: *A*_2_, {corrective maintenance}: *A*_3_, {immediate maintenance}: *A*_4_. Regard a series of historical health reliability degree as evidence. First, obtain the series of experimental data and the previous seven series. The historical health parameters are shown in Table 9 and are fused by three experts’ decision methods. Deal with data by grey processing to obtain the grey interval and calculate separately. Deal with data by grey processing to obtain the grey interval and calculate the confidence of each level separately. The decision result of evidence 1: historical health trends are shown as (15).

According to detection conditions, the historical maintenance number is selected as five and the total experiment time is 100 times. The next inspection time is set as 300 days and the overhaul cycle is 365 days. Attribute weights are determined as ω = (ω_1_, ω_2_, ω_3_) = (0.7719, 0.05, 0.1781). ω_2_ = 0.05 is the maintenance probability, which is equal to historical maintenance times/total test times. ω_3_ = 0.1781 is the overhaul rate, which is equal to the next inspection time/overhaul cycle. In the example, the historical maintenance times are five and total test times are 100. The next inspection time is 300 days and the overhaul cycle are 365 days. The decision matrix that was established by the fusion of each decision method is shown from Table 10, Table 11 and Table 12. The attribute weights of these three decision methods are (0.4158, 0.3467, 0.2375), which is attained by (10)~(12). The whitening degree is the mathematical expression of whitening rules in the grey set under the existing information. Whitening degree is obtained by whitening function, which is shown in Figure 7. The comprehensive decision matrixes that were obtained using the three decision methods by using (14).

**Figure 12:**
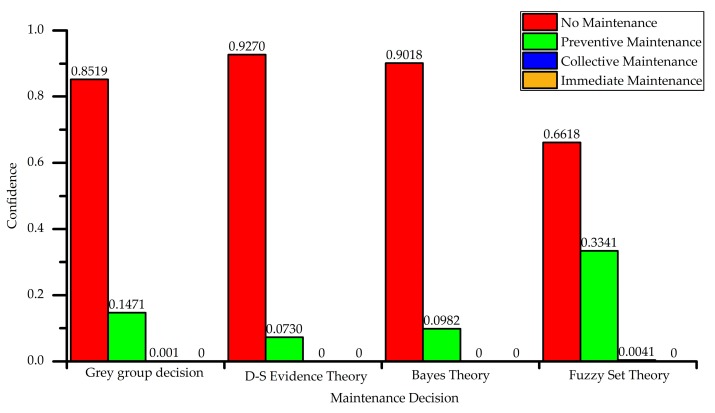
The confidence to 4 health status level of Situation 1 based on 4 decision methods.

Since the results of the matrix are floating from 0 to 1, normalization is not required. The final grey group decision-making result can be obtained by directly fusing with the weight. For another decision-making method, such as D-S evidence theory, Bayes theory, and fuzzy set theory are used as the off-the-shelf maintenance decision-making method. The decision results are shown in Figure 12. According to descending order of the maintenance confidence, the ranks and maintenance suggestions that are based on grey group decision and another three comparative decision methods are shown in Table 13, the rank of four methods are shown as *A*_1_ > *A*_2_ > *A*_3_ > *A*_4_, the final maintenance suggestion of four methods are all *A*_1_: no maintenance, which is suitable to the status description.

**Figure 13:**
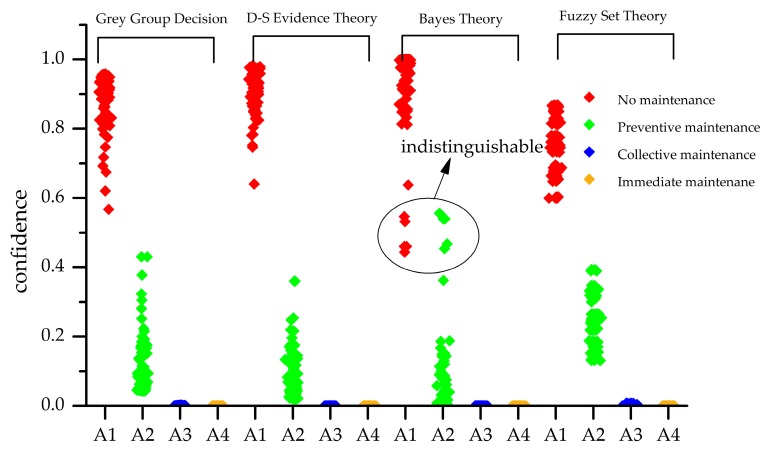
The confidence of 100 groups health state data to different health status level.

In order to verify the evaluation ability of maintenance decision method for health status, 100 groups of health status data at different times were used to test the accuracy of evaluation. The confidence of the 100 groups health state data to four health status level are shown in Figure 13. False alarm occurred when using Bayes theory. The accuracy for the four methods is shown in Table 14. Grey group decision ignores the disadvantage of Bayes theory for this situation to improve the decision accuracy.

**Situation 2: single sensor is failure**

To verify the effectiveness of the grey group maintenance decision method, situation 2 is used to verify the versatility and correctness of the method. In situation 2, the data of each group are all kinds of failures simulated by fault simulation software. The attribute weights are ω = (ω_1_, ω_2_, ω_3_) = (0.0932, 0.4, 0.5068). In this example, the historical maintenance times are 40 and the total test times are 100. The next inspection time is 250 days and the overhaul cycle is 365 days.

**Figure 14:**
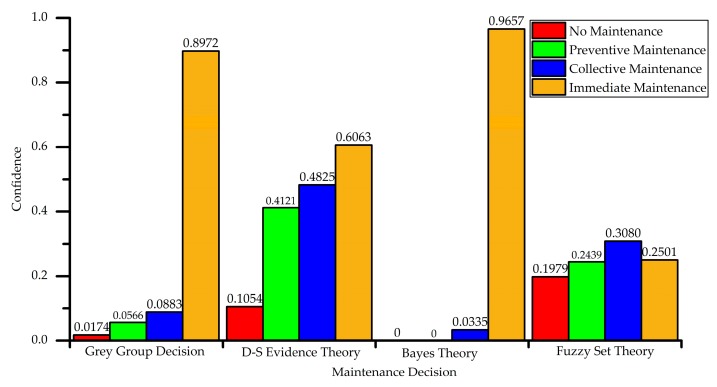
The confidence to 4 health status level of Situation 2 based on four decision methods.

The decision results of four methods are shown in Figure 14. All of the decision results are immediate maintenance except for the fuzzy set. The ranks and maintenance suggestions based on grey group decision and other three comparative decision methods are shown in Table 15.

**Figure 15:**
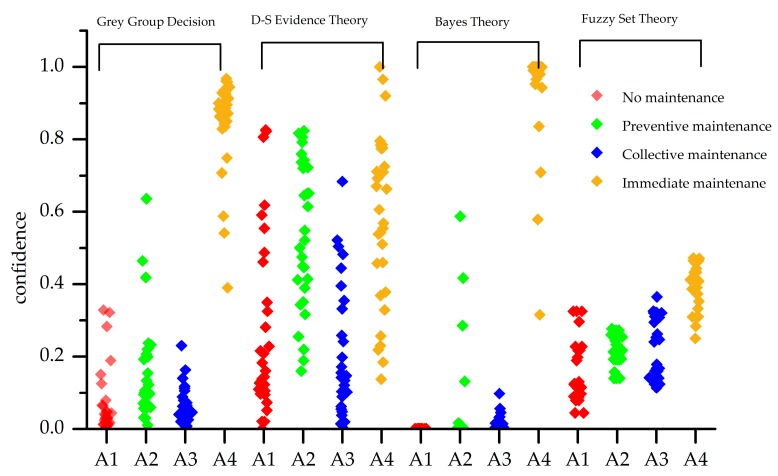
The decision result of 100 group failure state data.

To verify the effectiveness of the grey group maintenance decision method for single failure. 100 groups of failure status data are used for testing. The decision results are shown in Figure 15. The decision accuracies are shown in Table 14. The result of grey group decision is the same as Bayes theory and it is superior to the other algorithm. The result of D-S evidence theory is fluctuated and it is hard to distinguish the optimal maintenance result. The confidences of four health status level of fuzzy set theory are almost at the same level and it is difficult to realize the optimal maintenance result Grey group decision utilizes the advantage of Bayes theory for this situation to ignore the inaccuracy result of the other experts.

The accuracy of maintenance decision-making which is verified by 400 groups different health status level samples (100 groups for each health status level) are shown in Table 14. It is not difficult to find out from the above test that D-S evidence theory has good detection results at health state. However, it cannot be detected effectively at the failure state because of the large range data fluctuation. Bayes theory has a good decision-making result for the failure status, but false alarm will appear at health state. The fuzzy set theory is very good in the health state. But, in the case of failure, the health statuses often appear with a similar confidence, and cannot be effectively evaluated. According to the group decision method, the grey group decision exerts the advantage of the D-S evidence theory and fuzzy set theory in the health state and it reduces the missing alarm rate. In the case of failure, it plays the advantage of the Bayes theory and it reduces the probability of false alarm.

## 5. Conclusions

In this paper, a method of health management decision strategy of a sensor system is proposed by utilizing *HRD*-*GGD* theory. Health reliability degree strategy is utilized to quantify system state and to provide support for decision making. The system can provide the maintenance suggestion after the system runs and give the confidence degree of each maintenance proposal. The experimental results show that this method can evaluate the system state effectively, and the accuracy rate of maintenance recommendation is 98.25%. The result proves that the accuracy is improved over 2% when compared with the other methods and the decision results are optimal under all health status levels.

In the future, we will investigate the remain life of sensor system and failure prediction by analysis of the historical trend of MOS sensor degradation in the sensor system.

## Figures and Tables

**Table 1:** Maintenance level of health and maintenance decision fault preventive measures.

Solution Set	Health Status Level	Health Description	Maintenance Level
*A*_1_	Health (*HS*)	healthy condition	No maintenance
*A*_2_	Subhealth (*SHS*)	normal range	Preventive maintenance
*A*_3_	failure edge (*FES*)	fault edge	Corrective maintenance
*A*_4_	Failure (*FS*)	fault condition	Immediate maintenance

**Table 2:** Health status level.

Solution Set	Range of Health Reliability Degree	Health Status Level
*A*_1_	0.9 ≤ *HRD* ≤ 1	Healthy
*A*_2_	0.6 ≤ *HRD* < 0.9	Subhealthy
*A*_3_	0.2 ≤ *HRD* < 0.6	Failure edge
*A*_4_	0 ≤ *HRD* < 0.2	Failure

**Table 3:** *HRD* computing procedure.

*HRD* Based on Grey Theory
**Input:** Output of the sensor system **Output:** Health Reliability Degree **Procedure:** Step 1: Establish the grey evaluating criterions, which is shown as (HS, SHS, FES, FS). Step 2: Determine the whitening function of the grey model according to Equations (1)–(4). Step 3: Compute decision weights by using information entropy method. Step 4: Compute Grey Sample Evaluating (*GSE*) Matrix by (5). Step 5: Calculate the *CGAV* under evaluating criterion sets. Step 6: Calculate *HRD* by RVM.

**Table 4:** The comprehensive decision-making step of grey group decision-making theory.

Grey Group Decision-Making Algorithm
**Input:** Historical Health Reliability Degree (*HHRD*): The parameter is composed of the last n *HRD*s. Maintenance Probability (*MP*): Maintenance probability is equal to history maintenance times/total test times. Overhaul Rate (*OR*): Overhaul rate is equal to the next inspection time/overhaul cycle. **Output:** Decision Result: The parameter is the level of the maintenance decision-making. The size of the framework is four: {no maintenance, preventive maintenance, corrective maintenance, immediate maintenance} Confidence: The parameter is output vector of the maintenance decision-making confidence. **Procedure:** Step 1: Calculate the interval grey numbers of each decision result for each evidence in the decision framework under each decision method. The interval grey number is expressed as $a_{ij}^{s}(⊗)$, $a_{ij}^{s}(⊗)\in [{\underline{a}}_{ij}^{s},{\bar{a}}_{ij}^{s}]$. ${\underline{a}}_{ij}^{s}$ represents the grey number lower limit and ${\bar{a}}_{ij}^{s}$ is the grey number upper limit (*i* = 1, 2, 3, 4, *j* = 1, 2, 3, *s* = 1, 2, 3). Step 2: According to the upper-lower limit $\left[{\underline{a}}_{ij}^{s},{\bar{a}}_{ij}^{s}\right]$ in the interval grey number $a_{ij}^{s}(⊗)$ in Step 1, establish the comprehensive decision matrix (CDM) of each decision method as shown in Table 5. Step 3: By utilizing the interval grey number weakening transformation, the decision matrix of three decision methods is initialized and transformed to obtain the standardized decision matrix. Step 4: Calculate the weight of each decision method. First, determine the effect vector of the three decision methods for all decision frameworks according to the standardized decision matrix in Step 3. The matrix elements are the effect vectors of the three decision methods for each decision result in the decision framework. Then, according to the interval grey number vector distance formula and the weight of each evidence attribute *ω_j_*(*j* = 1, 2, 3), the space projector distance of each effect vector is calculated. Finally, the ratio between the vector distance of a decision method and the sum of effect vector distance for the other decision methods is the weight coefficient *λ_s_*(*s* = 1, 2, 3, 4) of the decision method. Step 5: Calculate the comprehensive decision results. According to the maintenance decision result of the experts, the confidence of each decision result corresponding to the four decision frames can be obtained. Finally, according to the weight coefficients of each decision method that were obtained in Step 4, the final decision result is obtained by applying weighted averaging to the confidence.

**Table 5:** Comprehensive decision matrix of decision method $e_{s}$(*s* = 1, 2, 3).

	$u_{1}$		$u_{2}$		$u_{3}$	
$A_{1}$	${\underline{a}}_{11}^{s}$	${\bar{a}}_{11}^{s}$	${\underline{a}}_{12}^{s}$	${\bar{a}}_{12}^{s}$	${\underline{a}}_{13}^{s}$	${\bar{a}}_{13}^{s}$
$A_{2}$	${\underline{a}}_{21}^{s}$	${\bar{a}}_{21}^{s}$	${\underline{a}}_{22}^{s}$	${\bar{a}}_{22}^{s}$	${\underline{a}}_{23}^{s}$	${\bar{a}}_{23}^{s}$
$A_{3}$	${\underline{a}}_{31}^{s}$	${\bar{a}}_{31}^{s}$	${\underline{a}}_{32}^{s}$	${\bar{a}}_{32}^{s}$	${\underline{a}}_{33}^{s}$	${\bar{a}}_{33}^{s}$
$A_{4}$	${\underline{a}}_{41}^{s}$	${\bar{a}}_{41}^{s}$	${\underline{a}}_{42}^{s}$	${\bar{a}}_{42}^{s}$	${\underline{a}}_{43}^{s}$	${\bar{a}}_{43}^{s}$

**Table 6:** Procedure of calculating health status level.

Health Status Level
Step 1: to calculate the health level of the decision framework and the confidence of every evidence. Step 2: In grey group decision, the weight vector of each evidence attribute is ω = (ω_1_, ω_2_, ω_3_) Step 3: calculate the decision confidence for the expert set by ${\mathrm{confidence}}_{i}=\omega \cdot \mathrm{CDM}$. Step 4: the weight vector for each experts ω_e_ = (ω_e1_, ω_e2_, ω_e3_) is obtained by entropy weight method. Step 5: calculate the confidence of the final health status level by $\mathrm{confidence}=\sum_{i=1}^{3}\omega_{e}\cdot {\mathrm{CDM}}^{\left(i\right)}$.

**Table 7:** Failure type of senor system and its form.

Failure	Name	Failure Feature and Form	Failure Place	Failure Prevention and Control Measures
F1	Sensor disconnect	Step Response. Lower than lower threshold	Target gas sensor	Check the sensor pin, change the target sensor
F2	Sensor overload	Step Response. Above upper threshold	Target gas sensor	Check the sensor pin, change the target sensor
F3	Sensor poisoned	No response or irregular fluctuation	Target gas sensor	Change the target sensor
F4	Sensor drift	Slowly varying. Baseline offset	Target gas sensor	Increase the preheating time, change the target sensor
F5	Abnormal changed	Output fluctuation	Target gas sensor	Check and replace the filter capacitor, check and replace the power supply module, change the target sensor
F6	Heater circuit failure	Sensor has no response. Heater has no input.	Target gas sensor circuit	Circuit connection check, change the chips

**Table 8:** The scope of all the sensors.

Sensor	Range	Unit	Sensor	Range	Unit
CO-1	1–4	V	O_3_-1	0.15–1.8	V
CO-2	1–4	V	O_3_-2	0.15–0.9	V
CO-3	0.3–1.8	V	O_3_-3	0.15–0.4	V
NO_2_-1	0.3–5	V	SO_2_-1	2–5	V
NO_2_-2	0.3–5	V	SO_2_-2	1.3–5	V
NO_2_-3	0.1–1.5	V	SO_2_-3	1.3–3.7	V
T	15–50	°C	H	20–65	%RH
P	0.09–0.12	Kpa			

**Table 9:** Historical health parameters for situation 1.

Evidence	*A*_1_	*A*_2_	*A*_3_	*A*_4_
1	0.8150	0.3930	0.0039	0
2	0.8648	0.3357	0.0029	0
3	0.8568	0.3447	0.0029	0
4	0.8556	0.3457	0.0031	0
5	0.7337	0.4792	0.0057	0
6	0.9388	0.2373	0.0014	0
7	0.9393	0.2449	0.0014	0

**Table 10:** Comprehensive decision matrix of decision method $e_{1}$.

	*u*_1_		*u*_2_		*u*_3_	
	Grey Interval	Whitening Degree	Grey Interval	Whitening Degree	Grey Interval	Whitening Degree
*A*_1_	[0.8562, 0.8617]	0.8589	[0.8690, 0.8707]	0.8698	[1, 1]	1
*A*_2_	[0.1383, 0.1438]	0.1411	[0.5853, 0.5920]	0.5937	[0.1650, 0.1683]	0.1667
*A*_3_	[0, 0]	0	[0, 0]	0	[0, 0]	0
*A*_4_	[0, 0]	0	[0, 0]	0	[0, 0]	0
weight	0.7719		0.05		0.1781	

**Table 11:** Comprehensive decision matrix of decision method $e_{2}$.

	*u*_1_		*u*_2_		*u*_3_	
	Grey Interval	Whitening Degree	Grey Interval	Whitening Degree	Grey Interval	Whitening Degree
*A*_1_	[0.8860, 0.9026]	0.8943	[0.8690, 0.8707]	0.8698	[1, 1]	1
*A*_2_	[0.0974, 0.1140]	0.1057	[0.5853, 0.5920]	0.5937	[0.1650, 0.1683]	0.1667
*A*_3_	[0, 0]	0	[0, 0]	0	[0, 0]	0
*A*_4_	[0, 0]	0	[0, 0]	0	[0, 0]	0
weight	0.7719		0.05		0.1781	

**Table 12:** Comprehensive decision matrix of decision method $e_{3}$.

	*u*_1_		*u*_2_		*u*_3_	
	Grey Interval	Whitening Degree	Grey Interval	Whitening Degree	Grey Interval	Whitening Degree
*A*_1_	[0.6579, 0.6611]	0.6595	[0.8690, 0.8707]	0.8698	[1, 1]	1
*A*_2_	[0.3348, 0.3381]	0.3364	[0.5853, 0.5920]	0.5937	[0.1650, 0.1683]	0.1667
*A*_3_	[0.0040, 0.0041]	0.0040	[0, 0]	0	[0, 0]	0
*A*_4_	[0, 0]	0	[0, 0]	0	[0, 0]	0
weight	0.7719		0.05		0.1781	

**Table 13:** The rank and maintenance suggestion of Situation 1.

Method	Rank	Maintenance Suggestion
Grey Group Decision	*A*_1_ > *A*_2_ > *A*_3_ > *A*_4_	*A*_1_: No Maintenance
D-S evidence Theory	*A*_1_ > *A*_2_ > *A*_3_ > *A*_4_	*A*_1_: No Maintenance
Bayes Theory	*A*_1_ > *A*_2_ > *A*_3_ > *A*_4_	*A*_1_: No Maintenance
Fuzzy Set Theory	*A*_1_ > *A*_2_ > *A*_3_ > *A*_4_	*A*_1_: No Maintenance

**Table 14:** The accuracy of maintenance decision-making.

Health Status Level	Grey Group Decision	D-S Evidence Theory	Bayes Theory	Fuzzy Set Theory
*A*_1_	100%	100%	94%	100%
*A*_2_	100%	93%	100%	94%
*A*_3_	95%	40%	95%	60%
*A*_4_	98%	49%	98%	93%
average	98.25%	65.5%	96.75%	85.75%

**Table 15:** The rank and maintenance suggestion of Situation 2.

Method	Rank	Maintenance Suggestion
Grey Group Decision	*A*_4_ > *A*_3_ > *A*_2_ > *A*_1_	*A*_4_: Immediate Maintenance
D-S evidence Theory	*A*_4_ > *A*_3_ > *A*_2_ > *A*_1_	*A*_4_: Immediate Maintenance
Bayes Theory	*A*_4_ > *A*_3_ > *A*_2_ = *A*_1_	*A*_4_: Immediate Maintenance
Fuzzy Set Theory	*A*_3_ > *A*_4_ > *A*_2_ > *A*_1_	*A*_3_: Collective Maintenance
